# Breakdown of continuity in public mental healthcare in the Netherlands: a longitudinal case study

**DOI:** 10.5334/ijic.648

**Published:** 2011-09-16

**Authors:** André I Wierdsma, Meta van der Schee, Cornelis L Mulder

**Affiliations:** Department of Psychiatry Erasmus MC, O3 Research Centre Mental Healthcare Rijnmond, PO Box 2040, 3000 CA Rotterdam, The Netherlands; Municipal Health Department Rotterdam-Rijnmond, PO Box 70032, 3000 LP Rotterdam, The Netherlands; Erasmus MC, Teacher in BavoEuropoort Centre for Mental Healthcare, and Program Coordinator O3 Research Centre Mental Healthcare Rijnmond, PO Box 2040, 3000 CA Rotterdam, The Netherlands

**Keywords:** continuity of care, admission follow-up, public mental healthcare

## Abstract

**Introduction:**

Continuity of care for long-term service-dependent patients in the public mental health system requires intensive collaboration between all agencies involved. Understanding the ways in which various aspects of continuity of care interact may reveal help to find out more about how care delivered over time improves outcomes.

**Case study:**

Based on medical records, an addicted couple was monitored for number and type of contacts with health and social services. Over the years, 81 social workers or nurses, spread over 25 health and social services, have been involved in the rehabilitation process. Breakdown of continuity of care is linked to lack of information, missing procedures and guidelines, fragile relationships with the patient, and a reluctant public health approach.

**Conclusion:**

Prominent among relevant factors is the absence of protocols governing the transfer of patients between the various links in the continuum of mental healthcare services. High-quality follow-up after admission is partly a matter of professional principle in ensuring that problems in the chain of services are discussed. Case presentation in psychiatric journals should give systematic attention to sources of error in continuity of mental healthcare.

## Introduction

Continuity of care is associated with diagnostic accuracy, medication adherence, and reduced hospitalisation, and has been on the global political and social agenda for many years [1, 2]. The American Institute of Medicine has declared continuity of healthcare to be a primary aim in for improving healthcare quality and the American College of Physicians has placed it at the heart of far-reaching ideas for changes in service delivery [3]. Continuity of care has been identified by the UK’s National Health Service as a priority theme for its national Service Delivery and Organisation R&D Programme [4]. According to a report published in 2004 by the Health Council of the Netherlands, continuity of care is one of the basic principles of mental healthcare for severely ill patients in acute need, who often avoid contact with health services [5]. In the Netherlands, the increase in the number of compulsory admissions has led to the continuity-of-care concept being placed at the centre of the public mental health arena. In an attempt to reduce a ‘revolving-door’ phenomenon in community-based mental healthcare, outpatient follow-up of patients admitted involuntarily has become increasingly important [6]. The third national evaluation committee for the Dutch law on compulsory admissions referred to the importance of continuity of care, arguing that the principle of reciprocity requires a parallel obligation to provide appropriate health and social services, including ongoing care following discharge from compulsion [7].

Although continuity of care is considered important in today’s healthcare systems, the concept has been criticised for its lack of clarity. Generally, continuity of care is interpreted as the degree to which episodes of treatment are linked in a seamless, uninterrupted whole, in accordance with patients’ needs [8, 9]. However, healthcare workers from various domains have emphasised specific types of continuity, e.g. personal continuity highlighted in the doctor-patient relationship in primary care. Consensus is growing within the care sector that continuity is a multi-dimensional concept, involving information exchange, disease management, personal relationships, and flexibility of contact [1, 4, 10–14]. Understanding the ways in which these types of continuity of care interact, may reveal more about the mechanisms by which care delivered over time improves outcomes.

Without a better understanding, the mechanisms that affect continuity of care interventions may be misdirected or inappropriately evaluated [1]. Based on medical records in a case study spanning several years, this paper explores some of the problems that stand in the way of more effective continuity of mental healthcare.

## Case study

In 2000, Dutch television broadcasted a documentary about people who neglect themselves and their social environment. The film was shot in Rotterdam, one of the world’s largest seaports and the Netherlands’ second largest city with a population of approximately 550,000, a total of 1.2 million across the greater urban area. The documentary shows public mental health workers visiting people who fall outside the standard healthcare system. These people are at the fringes of society and include a man from a wealthy family living in a faeces-stained apartment, an old organ grinder sharing living and working space with a heroin-addicted prostitute, a mentally-disordered woman who keeps hundreds of mice as pets, an old and lonely opera singer who rarely leaves home, and a (formerly) substance-addicted couple who have lost parental rights. The present study follows up on the couple’s case (names have been omitted to ensure privacy; the couple have given written consent).

The addicted woman and her partner, both older than 40 and living together as an unmarried couple, have German and Italian nationality respectively. They have been living in the Netherlands since the 1980s, after being prosecuted in Italy and expelled from Germany because of drugs-related problems. The man found employment in Rotterdam and arranged for the woman and their newborn son to join him. At the time the documentary was filmed, their son was about eleven years old and had been living with a foster family for some years. Their daughter, just over one year, was placed in foster care by a juvenile court on request of the Council for Child Protection. Because of staggering debts the couple moved house repeatedly; through an alcohol and drugs clinic they rented an ill-maintained and sparsely furnished apartment in the private sector. Most likely because of coke abuse the patient suffered a heart attack. Her partner called for help when she had the attack, but because of language problems the local ambulance service did not respond immediately. In a comatose state the patient was taken to a general hospital, where a specialist diagnosed that she had an anterior wall myocardial infarct. After ten days of artificial respiration in the intensive care unit, the patient woke up from her coma and recovered well physically. However, her mental condition did not improve (possibly differential diagnosis: brain damage because of lack of oxygen after cardiac arrest).

This marked the starting point of a long journey through various health and social services. A document study showed that over a period of years no less than 81 social workers or nurses, spread over 25 health and social services, have been involved in the case. Dutch healthcare consists of a mix of public and private services in connection with a mixed system of insurance and healthcare funding. Most of the specialist care is organised by hospitals, which are managed on a private non-profit basis. General practitioners mainly work in private practices and play an important gate-keeping role in access to specialist services. Social welfare services constitute an adjoining set of arrangements to support people who have social or financial problems. Although there have been many changes in the Dutch healthcare system in recent years, medical and social welfare services on the whole have been free from charge at point of use and readily available. Despite the availability of healthcare and social welfare services, there is a ‘multiple-problem group’ that does not use these facilities. Local Health Authorities and private services play a role in public mental healthcare aiming to bring care-avoiding patients into the mental healthcare system. This includes healthcare and social support for drug addicts. Local services and support programmes take a medical perspective on addiction in an effort to avoid forced hospitalisation or imprisonment.

Healthcare providers in the Netherlands are obliged by law to set up a procedure to handle complaints and to install a committee that records and investigates medical errors and near accidents. Public mental healthcare, however, is characterised by a supply-chain approach so that it is often unclear which part of the chain is responsible for a particular error or near accident. It is not the intention of this case study to point a finger at one specific part in the chain of services, because there are no simple solutions for complex problems. We link the breakdown of continuity of care to (1) lack of information, (2) missing procedures and guidelines, (3) fragile relationships with the patient, and (4) a reluctant public health approach.

## Breakdown of continuity of care

### Lack of information

Information continuity is the transfer of information that links the care provided between healthcare events [1, 15]. Ideally, at first contact, the healthcare specialist would make a comprehensive, independent and objective assessment of the patient’s needs. Information continuity implies accumulating knowledge of a patient’s illness, history, treatment complications, and social circumstances. Information continuity also involves transferring information from one service to another while excluding or limiting informal, undocumented information. Detailed information also aids in the creation of informed assessments of the acuteness of the circumstances.

The patient was stealing from other patients, and because of her behaviour she could no longer stay on the cardiology ward. The general hospital asked the Local Health Authority for an assessment for a follow-up admission to a nursing home accommodation for the homeless. According to the Authority, however, referral to services for the homeless was not the most obvious route to take because the patient had a home. Moreover, it became clear that alternatives, such as admission to a psychiatric hospital, had not been considered. The patient was then examined by a psychiatrist allied to the hospital, who made a provisional diagnosis of personality disorder, intensified by substance abuse, and paranoid characteristics. Gradually the patient’s physical and mental condition deteriorated: she lost weight and withdrew into herself, alternated by aggressive moods. The patient displayed wandering behaviour, and several times she was found on the streets in a state of catatonia. These states increased in frequency and at the end of 2002 a compulsory admission was arranged by an emergency service. At first, the patient was in a psychiatric hospital anonymously because it was not possible to talk with her. Because of aggressive behaviour, she had been isolated for some days, and she was subsequently transferred to a nursing department.

The general hospital in this case had a primary-task-focused view, whereas a wider perspective may help to link healthcare episodes over time and between services, such as primary healthcare, social services, housing corporations and employment agencies. Although an ‘old acquaintance’ in public mental healthcare, the patient was not known to the emergency services and the psychiatric hospital. Modern electronic systems could potentially contribute to better continuity of information by informing all involved parties of identifying characteristics of anonymous patients (e.g. claw hand deformity caused by nerve damage through injecting intravenously). However, a real-time electronic health record system accessible to relevant staff across providers was not in place. As in other European countries, information technology was not implemented as smoothly as expected and information-based approaches to improve healthcare services remain a long way off [16]. Patient-held shared care records offer an alternative basis for communication between patient and service provider and among all agencies involved. In mental healthcare, however, use of patient-held records is targeted to very specific patient groups and settings, e.g. joint crisis plans and use of compulsory treatment [17].

Lack of information and inability to transfer information across services affect other levels in the continuity framework, e.g. management continuity and relational continuity of mental healthcare. Medical and social details, including social support mechanisms and personal idiosyncrasies (norms, values and expectations), not only improve the quality of care plans, but also ensure that patients feel listened to and understood.

### Missing procedures and guidelines

Management continuity is conceptualised as a consistent approach based on a comprehensive treatment plan that, when necessary, focuses on transfer between services according to service standards and protocols [1, 15]. Complex, often chronic, health problems require care from a variety of services and this care must be based on shared management plans or care protocols in order to prevent providers from acting inconsistently. However, in the absence of ‘care pathways’ in which the content and timing of interventions are prescribed, health and social services can transfer patients to other service sectors without deciding on a healthcare plan in consultation with other appropriate stakeholders.

The general-hospital psychiatrist reported that the patient showed impaired judgement/disordered thinking, lack of insight, and lack of illness awareness. There was loss of memory to the extent that the patient had forgotten that she had given birth to a daughter and had formerly used drugs (presently no craving). The psychiatrist concluded that there was no primary psychiatric disorder, and also no ground for compulsory admission. The patient was incontinent, and dependent on social support, but was discharged from the general hospital without adequate care being in place to support her.

Complexities of government rules and regulations, and lack of efficient coordination, contribute to the breakdown of continuity of care. Legal matters and specific social care arrangements can contradict one another.

Because of vermin and drugs misuse and dealing, the patient’s home was put on the ‘nuisance premises list’, and a so-called Mayor-closure was executed. However, the local authority failed to provide accommodation elsewhere. In a neighbouring municipality new accommodation was arranged through the community-care network (a collaboration of primary care services). In their new hometown, the couple’s problems accumulated faster than solutions could be found. When Rotterdam ended their supplementary benefit, the local social services did not initially take over owing to suspicion of illegal employment and unclear immigration status. Living expenses were not paid for several months, as a result of which there was a constant threat of water and gas shut off and eviction. The national office for collecting parental contributions was quick to send in the bailiffs for a claim of several thousand euros and almost immediately a representative from the judicial collection agency followed concerning unpaid fines for drunk driving and for illegal use of public transport.

Discharge planning, rapid transfer between services, and implementation of individualised care plans are key characteristics of management continuity [18]. In the Netherlands, however, there are few quality criteria for hospital discharge. Even in the case of emergency compulsory admission, the medical director himself is authorised to discharge a patient, or to discontinue the coercive measure when the patient shows the necessary willingness for treatment, and admission is continued voluntarily. The front-end of compulsory admission procedures is structured through regulations and forms, but the follow-up after admission is not as well organised [19].

Soon after the patient had been involuntarily admitted, the hospital stay became problematic as it proved difficult to take corrective measures, and her behaviour did not seem to be affected by medication (chlorprothixene). No follow-up compulsory measure had been applied for, so that after expiration of the emergency compulsory admission her stay was on a voluntary basis, and she could leave the hospital at any time. At the urgent request of the Local Health Authority, neuropsychological tests were administered to gain an insight into the patient’s physical and cognitive decline. Without consulting test results or informing her partner, the patient was discharged from the psychiatric hospital and was taken home by taxi. However, she was left at her front door with only a refuse bag for a raincoat. The hospital discharge letter was addressed to her former general practitioner, although it should have been clear from the information available that there had been no contact for several years. In effect, no general practitioner, and no transfer to outpatient mental healthcare had been organised. In mid 2003 a local platform investigating problems of coordination in public mental healthcare discussed this matter under the header ‘Mrs. Murphy’. In response to remarks by the head of the Local Health Authority, the psychiatric hospital stated that the usual procedures had been followed, and suggested that the patient could take up the matter with the hospital’s committee of complaints.

Because of lack of quality standards, it is difficult to evaluate outcome measures, such as length of stay or the time interval between hospital discharge and follow-up. Moreover, because aftercare is not regulated, there is no professional panel to call to account individual health and social services on for their performance.

Other types of continuity of care are supported by well-developed management continuity. Care pathways and individualised care plans structure the knowledge accumulating on the patient, facilitate the active involvement of patients in their own care and treatment plan, and specify the scope of the actions to be taken, giving both provider and patient something to hold on to [2, 18]. Without this type of continuity, healthcare professionals will often find it difficult to justify the time and effort needed to build up trustful relationships and sustain contact with patients who refuse the help they need.

### Fragile relationships

Relational continuity involves an ongoing therapeutic relationship with healthcare professionals who will respond on a personal level in a setting that promotes cooperation and a coordinated, inter-subjective approach [1, 15]. Concentrating care with a few medical and social workers is essential to building a trustful relationship and supporting mutual understanding. As well as helping accumulate the medical and contextual knowledge that improves care plans, relational continuity encourages a sense of responsibility towards patients. This sense of responsibility should be extended so as to include those who attend the discussion of the treatment plan with, or for, the severely ill patient [20]. Without this responsibility for patients and families, continuity of care can be easily set aside.

The alcohol and drug clinic stopped treatment because the patient no longer belonged to the target group, and her partner failed to keep his appointments. Efforts to find care arrangements elsewhere failed because, for example, the homecare service considered the task not feasible. However, the patient’s need for care did not decrease. Social workers repeatedly noticed a lack of food or drink in the house. Yet, because of several changes of personnel owing to ill health or career moves, it was months before the local debt help service was able to start a financial governance procedure to gain control over the couple’s spending. In a meeting of all parties concerned, including the city councillors responsible, mental health services were pressed to start treatment and psychiatric homecare. The response, however, was hesitant because aftercare and financing were uncertain. A request for specialised homecare had been approved, but the patient refused to let social workers into her home when she was alone. In addition, it proved difficult to find a general practitioner accepting new patients. The nearby general practitioner’s office only reluctantly agreed to make a house call when the patient fell down and had to be taken to hospital with a severe eye injury. Again, the patient’s physical and mental condition deteriorated. In a temporary psychotic state and undernourished she was admitted voluntarily to a psychiatric hospital.

Impaired awareness of illness and lack of social support hinder severely mentally ill patients in influencing their care plan or in following procedures to issue complaints. In the context of compulsory admission, support for long-term service-dependent patients takes the form of legal aid. In Dutch law, however, legal aid extends only to the first phase of the admission; consequently, no one on behalf of the patient is watching the transition from involuntary admission to voluntary hospitalisation [21]. This is worrying as approximately 40%–50% of the patients do not know the legal status of their hospitalisation [22]. Without social support, the relationship between a severely ill psychiatric patient and healthcare services can become problematic. In the words of Bachrach [8]: continuity implies the availability of an enabler who will assist the patient in gaining access to the system.

Severely ill mental patients often need more than one service provider at a time, although many chronic patients find it difficult to maintain multiple relationships. This problem can be resolved through staff sharing between several providers, so that patients can relate to a ‘primary nurse’ in both inpatient and outpatient settings. An alternative solution is the team-treatment approach, whereby a combination of providers assumes responsibility for coordinating health care and social support. This approach also reduces the potential risks of long-term one-to-one relationships, such as breakdowns in communication due to violated norms, unmet expectations, or a mismatch of personality styles [8, 14].

The team-based approach has been implemented in the form of local community-care networks (CCNs) and assertive community treatment teams (ACTs). CCNs have a significant impact on the number of admissions, including compulsory admissions [23]. Likewise, ACTs have been introduced to prevent psychiatric admission and improve aftercare for severely mentally ill patients. Multi-disciplinary, outpatient ACT teams support patients on a 24/7 basis covering a range of problem areas [24]. Sustaining long-term contact is essential in this team-based public mental healthcare approach.

### Reluctant public health approach

Continuity of contact refers to sustaining long-term connected care in a coherent, interdisciplinary way by creating a service continuum to accommodate patients’ needs and by documenting outcomes and follow-up appointments [15]. Regular contact is a prerequisite not only for building a strong personal relationship, but also for ensuring that management goals are adapted in accordance with patients’ needs over time, and for monitoring the acuteness of patients’ problems and changes in circumstances.

During the patient’s voluntary admission, the son ran away from his foster home and moved in with his parents. The father viewed the son as an extra carer, but it soon transpired that the patient was being maltreated by her son. Childcare was fully informed, but did not intervene. It was difficult for the patient to understand that she could press charges, and her partner trivialised the abuse to avoid taking sides. An assessment procedure for sheltered living was started, but when the application finally came through, the couple rejected nearby accommodations, and chose to wait for a place to become available in another project. At the end of 2005, the couple moved back to sheltered living in Rotterdam. The son was placed in a boarding school, but in early 2006 he ran away and again moved in with his parents. The patient’s physical and psychological abuse continued.

Continuity of contact requires that mental healthcare organisations facilitate a broad range of services that must be in place to avoid unnecessary barriers to treatment access. Housing facilities, employment projects, and other rehabilitation projects are needed to create a stable environment that contributes to the recovery of severely ill patients [25]. However, this type of support is scarce. In Dutch mental healthcare the number of long-term service-dependent patients has increased steadily over the years [26]. An assertive outpatient approach can be a valuable tool for strengthening contact between treatment staff and severely mentally ill patients who often drop out of treatment programmes [27]. However, because of a reluctant public mental healthcare approach in this case, the couple continued to have personal freedoms that failed to create a more stable household.

## Discussion

Continuity of mental healthcare has been identified as a priority target for changes in service delivery. The problem is often linked to changes that have led the delivery of psychiatric services to become fragmented—mainly changes in adjacent service-delivery systems, eligibility criteria for accessing treatment programmes, and the creation of multiple funding streams. Traditionally, the doctor role combines assessment, diagnostic treatment, disease management, and maintenance of a caring relationship. However, this horizontal integration, captured in the patient-practitioner relationship, has evolved into a social system that links doctors to service users, supported by treatment teams and healthcare organisations, and monitored by health insurers and health authorities. All actors bring their role specific expectations, sanctions, norms, and values into the interaction processes. Modern society has created a complex, vertically integrated healthcare system and the continuity problems that come with it [28].

Types or dimensions of continuity of care refer to discrete actors in the processes that bring about continuous improvement in quality of care. To use the jargon of multilevel analysis: in a four-level structure, healthcare contacts are clustered within patients and patients are clustered within providers and healthcare districts. First, at district level, continuity of care implies an organised collection of medical and social information about each patient. Second, there should be an organised team of providers that assumes responsibility for the quality of care. Third, an ongoing relationship must be built on trust between the patient and a personal healthcare professional. Finally, at patient level, continuity of contact implies maintaining long-term connected care for severely ill patients in a coherent way, by creating a flexible service continuum, and documenting the process of rehabilitation [15].

This case study has highlighted a number of mechanisms that show a negative impact on better continuity of mental healthcare. After the first hospital admission for heart failure, it was evident that it would be very difficult, if not impossible, for the patient to live on her own. However, it took years to effect intensive tenancy support, and even then quality of life was impaired.

For long-term service-dependent patients, effective support hinges on the intensive cooperation of health and social services and creative handling of rules and regulations. A recurrent problem in this case history is the transfer between links in the chain of public mental healthcare. To counteract these problems, a comprehensive plan of action is needed. This should involve the development of high-quality information systems at district level, using standardised assessment instrument linked to service-delivery criteria and protocols at provider level, and a wide range of services and leverage types in order to arrive at a tailor-made approach at team level and patient level. Better information exchange and feedback are key-issues in this process, as are policies that structure decision-making for mental health professionals, integrate the actions of different services, and favour long-term care and transparency of service delivery.

However, although standards and pathways of care are important, high-quality follow-up after admission is in part a matter of professional principle in ensuring that problems in the chain of services are discussed. Despite all efforts, control mechanisms and procedures can break down, in which case errors should be reported and acted upon to prevent repetition. As in other areas of healthcare, reports on medical errors and near misses can help make this divided responsibility more transparent and communicate professionalism and good practice. Case presentation in psychiatric journals should not only concern creative diagnosis and treatment combinations, but also give systematic attention to sources of error in mental healthcare. In 2002, the American journal *Annals of Internal Medicine* started a new series of case presentations to highlight errors and near accidents, and general quality issues. The series-editors evaluated individual cases, based on the exploration of medical documents and interviews, and in some cases were invited by the hospital concerned to investigate internal quality procedures. In a case conference model, results were discussed with national experts on patient safety and quality control in order to focus on the general issues underlying the specific case presentations [29]. This type of transparency is worth reproducing in public mental healthcare.
